# Incomplete rather than complete nasolacrimal duct obstruction Is strongly associated with meibomian gland dysfunction in postmenopausal women with PANDO: a cross-sectional study

**DOI:** 10.3389/fmed.2026.1831157

**Published:** 2026-04-30

**Authors:** Haili Jin, Yin Liu, Xianjie Chen

**Affiliations:** Department of Ophthalmology, Wuhu Eye Hospital, Wuhu, Anhui, China

**Keywords:** association, meibomian gland dysfunction, postmenopausal women, primary acquired nasolacrimal duct obstruction, risk factors

## Abstract

**Background:**

Although postmenopausal women with primary acquired nasolacrimal duct obstruction (PANDO) frequently present with concurrent meibomian gland dysfunction (MGD), and PANDO is known to contribute to ocular surface inflammation, the specific factors driving MGD in these patients remain unclear. This study aimed to identify and assess key demographic, clinical, and hormonal factors associated with MGD.

**Methods:**

This prospective study included 272 postmenopausal women (180 with PANDO, 92 controls). All underwent standardized assessments: meibography (Keratograph 5 M) for gland loss, slit-lamp MG evaluation, and the Ocular Surface Disease Index (OSDI) questionnaire. Multivariable regression identified independent MGD risk factors, adjusting for age, menopause duration, obstruction completeness, disease duration, and dacryocystitis. Exploratory tear cytokine (*n* = 71) and serum hormone (*n* = 98) analyses were performed.

**Results:**

After adjustment, incomplete obstruction showed the strongest independent association with severe upper eyelid MG loss (95% CI: 2.48–8.82, *p* < 0.001) and a 2.02-fold increased odds of worse lower eyelid meibum quality (95% CI: 1.13–3.63, *p* = 0.019). A longer duration of PANDO was associated with reduced odds of expressibility impairment (OR = 0.96 per year, *p* = 0.027). Higher non-invasive tear meniscus height predicted longer tear breakup time (*B* = 2.85, *p* < 0.001). Several univariately significant factors lost independence in multivariable models.

**Conclusion:**

Incomplete PANDO is the principal factor associated with severe, often asymptomatic MGD in postmenopausal women in this cross-sectional analysis. Clinical evaluation should extend beyond epiphora. Routine meibography is recommended to detect silent gland atrophy and guide ocular surface preservation strategies.

## 1 Introduction

Ocular surface homeostasis depends on functional lacrimal drainage, which clears tears containing inflammatory mediators and pathogens (1). Primary acquired nasolacrimal duct obstruction (PANDO), a common idiopathic condition predominantly affecting postmenopausal women, disrupts this clearance and promotes ocular surface disease (2–4). Beyond causing epiphora and dacryocystitis, PANDO is associated with structural and functional damage to meibomian glands (MGs) (5–9) holocrine glands essential for tear film stability (7). Notably, meibomian gland dysfunction (MGD) often persists or worsens after successful PANDO treatment (8, 9), contributing to chronic ocular surface morbidity.

The convergence of PANDO and MGD is particularly salient in postmenopausal women, who face dual vulnerabilities: PANDO’s striking female predilection (10, 11) and age-related MGD exacerbated by hormonal changes (12–14). While demographic factors (e.g., age, menopause duration) and systemic hormonal status are established modifiers of MG function, their specific role in the context of PANDO remains poorly defined. Moreover, a critical and unaddressed question is whether the intrinsic clinical characteristics of PANDO itself such as obstruction completeness, disease duration, or the presence of dacryocystitis are the principal determinants of MGD severity. This question is particularly crucial because the prominent epiphora resulting from lacrimal drainage obstruction can overshadow sensations of dryness, complicating clinical assessment.

We therefore hypothesize that the phenotypic characteristics of PANDO itself, rather than patient demographics alone, are the primary drivers of MGs damage in this vulnerable population. Using comprehensive ocular surface assessment, we aimed to systematically evaluate a spectrum of potential risk factors with a specific focus on obstruction severity to identify the pivotal determinants of MGD in postmenopausal women with PANDO, thereby informing targeted prevention and refined clinical management.

## 2 Methods

### 2.1 Study design

**Figure 1:**
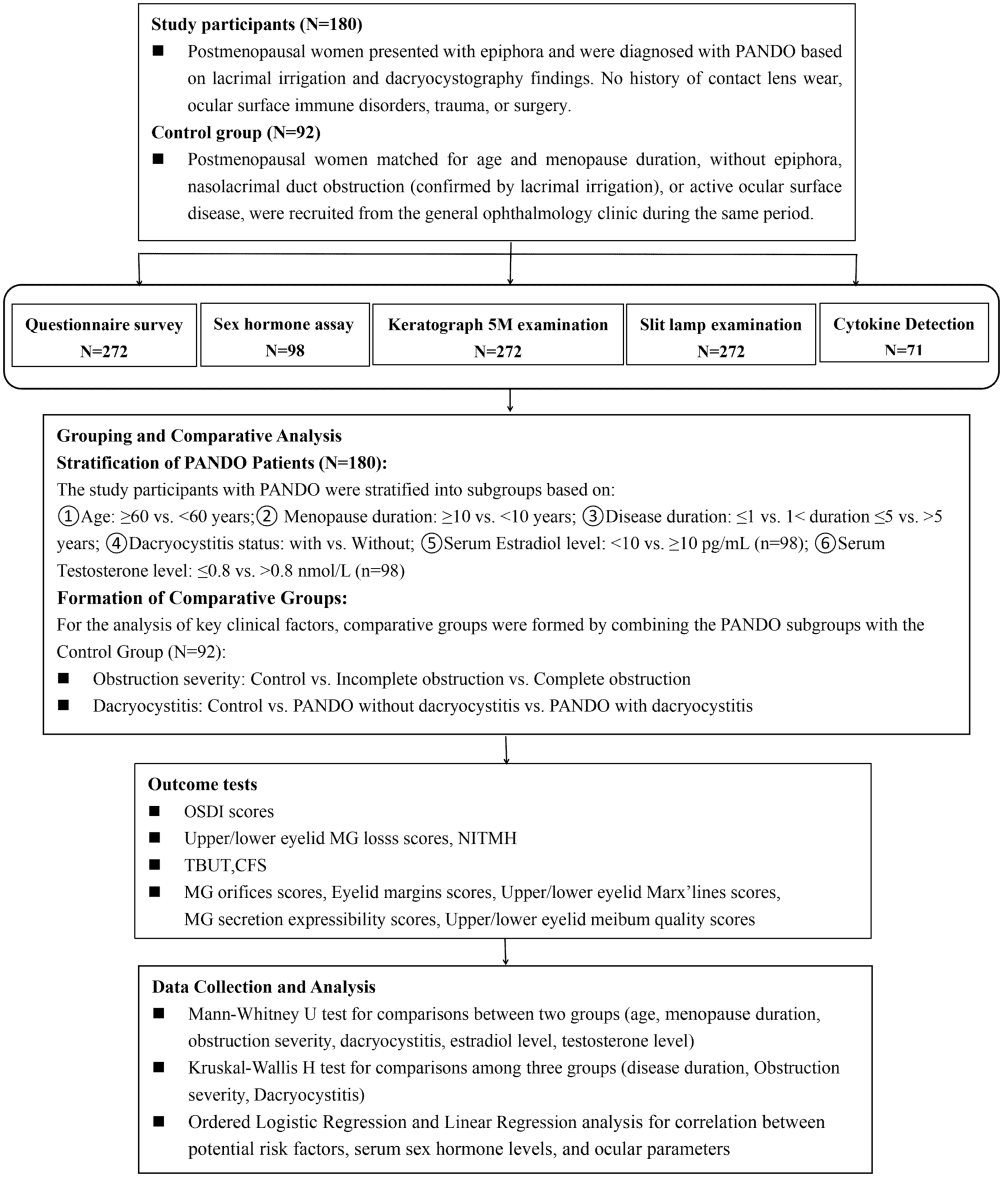
A schematic representation of the study design.

A schematic representation of the study design is provided in Figure 1. This cross-sectional study was conducted at Wuhu Eye Hospital, China (March 2021–February 2023), adhering to Declaration of Helsinki principles and approved by the Institutional Ethics Committee (No. 20210107). Written informed consent was obtained from all participants.

### 2.2 Participants

We enrolled 180 postmenopausal women with unilateral or bilateral PANDO, diagnosed by clinical epiphora assessment, irrigation testing, and CT-dacryocystography (15). Complete obstruction was defined as total reflux of irrigating fluid without passage into the nasal cavity or pharynx, representing complete anatomical occlusion of the nasolacrimal duct (NLD). Incomplete obstruction was defined as partial flow of irrigating fluid into the nasal cavity or pharynx with partial reflux, indicating residual lumen patency.

All diagnoses were confirmed by CT dacryocystography, which identified the obstruction as located in the NLD. Dacryocystitis was diagnosed by mucoid or purulent discharge upon irrigation without traumatic or secondary etiologies (16). 92 healthy volunteers, matched for age and duration of menopause and without epiphora, NLD obstruction (confirmed by lacrimal irrigation), or active ocular surface disease, were recruited from the general ophthalmology clinic during the same period. Exclusion criteria included: (1) Premenopausal status; (2) Systemic conditions affecting tear film homeostasis (e.g., Sjögren’s syndrome, diabetes); (3) Active ocular inflammation; (4) Prolonged topical medication use (≥3 months); (5) Contact lens use; (6) Ocular surgery or trauma history. For unilateral PANDO, the affected eye was studied; for bilateral cases, the more symptomatic eye was selected. Participants were stratified by age, menopause duration, disease duration, obstruction severity, dacryocystitis status, and serum hormone levels (estradiol [E2], testosterone [T]) in a subgroup (*n* = 98).

Baseline characteristics are summarized in Table 1. While participants were stratified by disease duration, dacryocystitis, obstruction severity, E2, and T levels, all inter-group comparisons were subsequently adjusted for differences in age and menopause duration.

**Table 1:** Baseline demographic and clinical characteristics of the study population.

Characteristics	*n* (%)
Demographic characteristics	
Age, *n* (%)	*N* = 180
Age < 60 years old	100 (55.56%)
Age ≥ 60 years old	80 (44.44%)
Menopause duration, *n* (%)	*N* = 180
Menopause duration < 10 years	100 (55.56%)
Menopause duration ≥10 years	80 (44.44%)
Relevant medical history	
Duration of the disease, *n* (%)	*N* = 180
Disease duration ≤ 1 year	54 (30.00%)
1 < Disease duration ≤ 5 years	63 (35.00%)
Duration > 5 years	63 (35.00%)
Obstruction severity	*N* = 180
Complete obstruction	78 (43.33%)
Incomplete obstruction	102 (56.67%)
With or without dacryocystitis	*N* = 180
With dacryocystitis	72 (40.00%)
Without dacryocystitis	108 (60.00%)
Sex hormone levels	*N* = 98
Estradiol < 10 pg./mL	67 (68.37%)
Estradiol ≥ 10 pg./mL	31 (31.63%)
Testosterone ≤ 0.8 nmol/L	47 (47.96%)
Testosterone > 0.8 nmol/L	51 (52.04%)

### 2.3 Meibomian gland and ocular surface evaluation

All examinations were performed under standardized conditions (9:00–11:00 a.m.; temperature: 22–28 °C; humidity: 40–50%) following a standardized sequence from non-invasive to invasive procedures. One ophthalmologist conducted all slit-lamp examinations and administered the Ocular Surface Disease Index (OSDI) questionnaire. A trained technician performed imaging using Keratograph 5 M (Oculus, Germany). MG loss and non-invasive tear meniscus height (NITMH) were assessed jointly by ophthalmologist and technician. MG loss was graded per eyelid on a scale of 0–3: 0 (no loss), 1 (<1/3 loss), 2 (1/3–2/3 loss), 3 (> 2/3 loss) (17). We selected gland dropout as a key metric because it is the most discriminative meibographic feature for MGD diagnosis. In the validation of reliable clinical grading scales, gland dropout achieved the highest area under the curve (AUC = 0.78), confirming its superior diagnostic ability compared to other lid margin signs (18). This objective imaging biomarker aligns with histopathological evidence of true glandular loss (19). Tear breakup time (TBUT) was measured using fluorescein strips (Tianjin Jingming New Technology Development Co., Ltd., China) and averaged over three trials (19). The non-invasive TBUT measurement on the Keratograph 5 M, which yields a shorter ‘first break-up time’ (NIBUT-first) compared to TBUT (20), was not used as the primary metric for tear film stability in this analysis, as recent evidence suggests TBUT may demonstrate better correlation with other clinical signs of dry eye in an aging population (20). Corneal fluorescein staining (CFS) was assessed across four quadrants (nasal superior/inferior, temporal superior/inferior), each scored 0–3 based on staining density and characteristics; quadrant scores were summed for a total CFS score (21). Marx’s line position was evaluated under cobalt blue filter using a three-segment eyelid margin division (medial, central, lateral), with each segment scored 0–3 based on its relationship to MG orifices. Total Marx’s line score was the sum of scores from all segments (22). Eyelid margin abnormalities (irregularity, vascular engorgement, orifice obstruction, mucocutaneous junction displacement) were evaluated under diffuse slit-lamp illumination. Each abnormality contributed one point to a total score (range 0–4) (23). MG orifice features (capping, narrowing, obliteration, etc.) were graded: 0 (normal), 1 (mild change), 2 (moderate obstruction/protrusion), 3 (severe obstruction/atrophy) (23). MG secretion expressibility was assessed by applying digital pressure to five central upper eyelid glands and scored as follows: 0 (all expressible), 1 (3 to 4 expressible), 2 (1 to 2 expressible), and 3 (none expressible) (24). Meibum quality was evaluated in eight central glands of each eyelid and graded: 0 (clear), 1 (cloudy), 2 (cloudy with debris), 3 (inspissated) (25). Serum estradiol (E2) and testosterone (T) levels were quantified using chemiluminescence immunoassays in a subgroup (*n* = 98).

### 2.4 Tear cytokine and hormone assessments

#### 2.4.1 Tear sample collection and cytokine analysis

For tear cytokine analysis, a case–control design was employed, enrolling 47 postmenopausal women with PANDO and 24 age-matched healthy postmenopausal women (total *n* = 71). Tear fluid was collected from all participants using Schirmer strips (Whatman 41# filter paper; Tianjin Jingming New Technology Development Co., Ltd., China) without anesthesia, following established methodologies (3). Strips with ≥10 mm wetting were eluted and analyzed for a panel of nine cytokines—Interleukin (IL)-6, IL-8, IL-1*α*, IL-1β, Macrophage Inflammatory Protein (MIP)-1α, Tumor Necrosis Factor (TNF)-α, and IL-10—via multiplex bead immunoassay (Luminex 200™) (3, 26).

#### 2.4.2 Serum hormone analysis

E2 and T levels were quantified in a subgroup (*n* = 98) using chemiluminescence immunoassays.

### 2.5 Statistical analysis

Data were analyzed using SPSS v31.0. Continuous variables are presented as median [interquartile range]. Non-parametric tests (Mann–Whitney U, Kruskal-Wallis H) were used for univariate comparisons. Multivariable analyses employed ordered logistic regression (ordinal outcomes) and multiple linear regression (continuous outcomes), with variance inflation factor examined for multicollinearity. Statistical significance was set at *p* < 0.05.

## 3 Results

### 3.1 Study population characteristics

This study enrolled 180 postmenopausal women with PANDO and 92 control subjects without PANDO. The groups were well-matched in age (60.80 ± 8.75 vs. 59.37 ± 5.95 years; *p* = 0.505) and menopause duration (10.58 ± 8.56 vs. 9.40 ± 6.83 years; *p* = 0.468). Among patients with PANDO (mean disease duration: 6.47 ± 8.09 years), the cohort was categorized according to age, menopause duration, disease duration, obstruction severity, dacryocystitis status, and hormone levels for subsequent analyses. Subgroup analyses for age, menopause duration, disease duration, estradiol, and testosterone included only patients with PANDO, whereas healthy controls were included only in analyses of obstruction severity and dacryocystitis. Baseline characteristics are detailed in Table 1. All comparative analyses were adjusted for age and menopause duration.

### 3.2 Alterations in ocular surface parameters: univariate analysis

To systematically evaluate factors associated with ocular surface damage, univariate analyses were first performed. As detailed in Table 2, advanced age (≥60 years) and prolonged menopause duration (≥10 years) were significantly associated with more severe MG loss in both upper and lower eyelids, worse MG orifice and eyelid margin abnormality scores, anterior displacement of Marx’s line on the upper eyelid, and a shorter tear film breakup time (TBUT) (all *p* < 0.05). Detailed results of the comparisons across age, menopause duration and duration of the disease groups are presented in Supplementary Tables S1–S3.

**Table 2:** Factors associated with ocular surface damage in postmenopausal women with PANDO.

Parameter	Age ≥60 vs. <60 years	Menopause duration ≥10 vs. <10 years	Disease duration ≤ 1 vs. 1 < duration ≤ 5 vs. > 5 years	Obstruction severity (complete vs. incomplete vs. control)	Dacryocystitis (with vs. without vs. control)	E2 < 10 vs. ≥10 pg./mL	T ≤ 0.8 vs. T > 0.8 nmol/L
Upper eyelid MG loss (score)	↑*(*p* = 0.016)	↑***(*p* < 0.001)	NS	↓***(*p* < 0.001)	↑*(*p* = 0.02)	NS	NS
Lower eyelid MG loss (score)	↑***(*p* < 0.001)	↑***(*p* < 0.001)	NS	↑*(*p* = 0.025)	NS	NS	NS
MG orifices (score)	↑**(*p* = 0.005)	NS	NS	↑*(*p* = 0.033)	↑*(*p* = 0.026)	NS	NS
MG secretion expressibility (score)	NS	↑**(*p* = 0.002)	NS	NS	NS	NS	NS
Upper eyelid meibum quality (score)	NS	NS	NS	NS	NS	NS	NS
Lower eyelid meibum quality (score)	NS	NS	NS	NS	NS	NS	NS
eyelid margins (score)	↑***(*p* < 0.001)	↑**(*p* = 0.005)	NS	↑***(*p* < 0.001)	↑**(*p* = 0.001)	NS	NS
Upper eyelid ML (score)	↑***(*p* < 0.001)	↑***(*p* < 0.001)	NS	↑***(*p* < 0.001)	↑*(*p* < 0.001)	NS	NS
Lower eyelid ML (score)	NS	NS	NS	NS	↑*(*p* < 0.028)	NS	NS
TBUT	↓*(*p* = 0.037)	↓*(*p* = 0.014)	NS	NS	NS	NS	NS
CFS	NS	NS	NS	NS	NS	NS	NS
OSDI (score)	NS	NS	NS	↑***(*p* < 0.001)	↑***(*p* < 0.001)	NS	NS
NITMH	NS	NS	NS	↑***(*p* < 0.001)	↑***(*p* < 0.001)	NS	NS

MG, meibomian gland; ML, Marx’s line; TBUT, tear film breakup time; CFS, corneal fluorescein staining; OSDI, ocular surface disease index; NITMH, non-invasive tear meniscus height; PANDO, primary acquired nasolacrimal duct obstruction; E2, Estradiol; T, Testosterone; NS, Not Significant; NA, Not available.

Symbols: ↑ Significantly increased in the first group listed (e.g., Age ≥60); ↓ Significantly decreased in the first group listed or versus the reference group (e.g., Control). The direction of arrows for “Obstruction Severity” and “Dacryocystitis” indicates the difference between the patient groups (Complete/With) and the Control group, unless otherwise specified by pairwise comparisons in the original data. *p < 0.05, **p < 0.01, ***p < 0.001.

Statistical methods: The Mann–Whitney U test was applied for comparisons between two groups (Age, Menopause Duration, Estradiol, Testosterone). The Kruskal-Wallis H test was applied for comparisons among multiple groups (Disease Duration, Obstruction Severity, Dacryocystitis), with significant overall *p*-values shown. Pairwise post-hoc comparisons with Bonferroni correction from original tables are summarized by the arrows and significance levels in the “Obstruction Severity” and “Dacryocystitis” columns.

**Figure 2:**
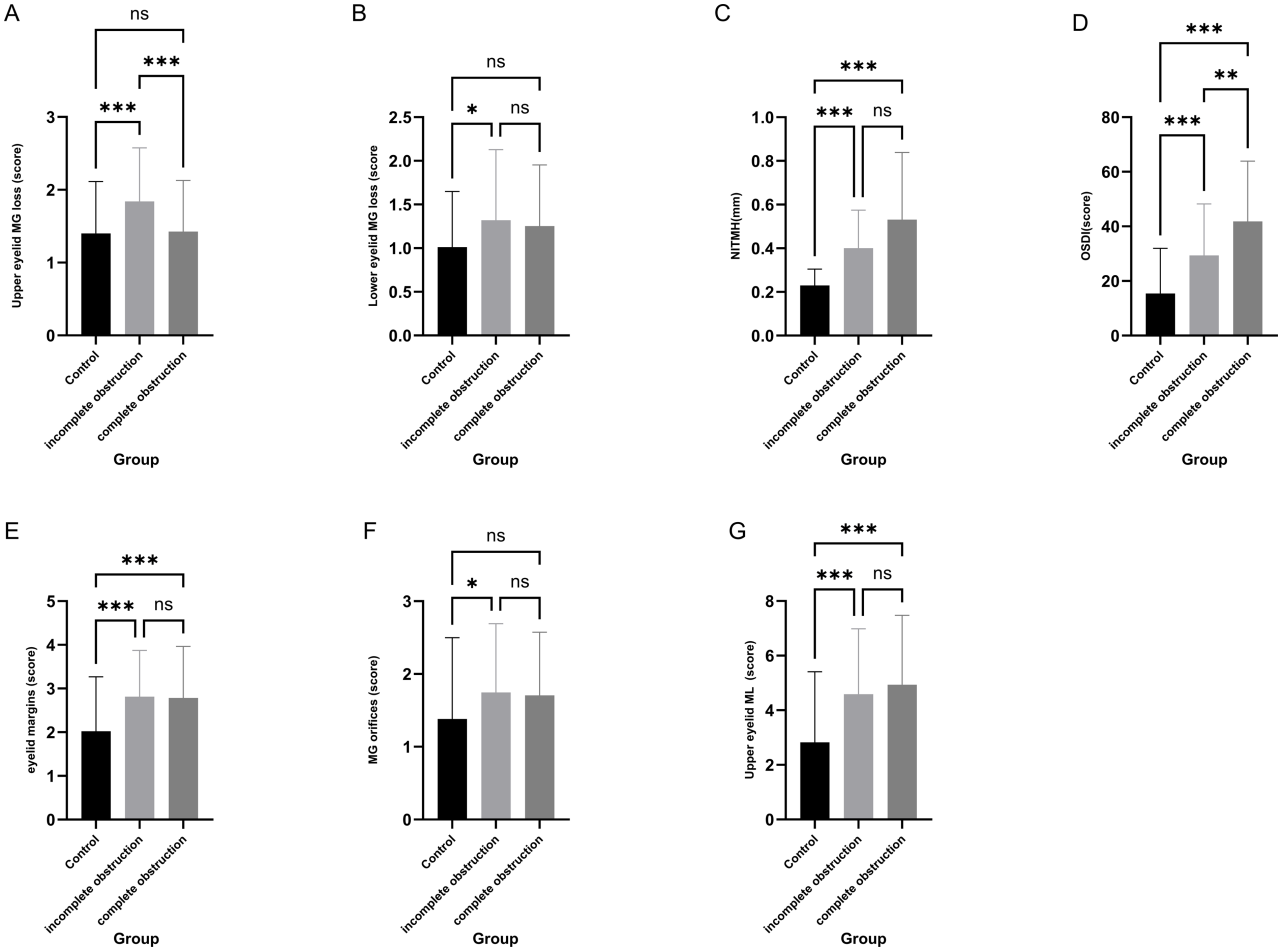
Alterations in ocular surface parameters associated with varying degrees of lacrimal duct obstruction. MG, Meibomian gland; NITMH, non-invasive tear meniscus height; OSDI, ocular surface disease index; ML, Marx’s line; Compared to controls, incomplete obstruction was associated with greater MG loss scores in the upper eyelid **(A)** and lower eyelid **(B)**, higher NITMH **(C)**, higher OSDI scores **(D)**, higher eyelid margin scores **(E)**, higher MG orifice scores **(F)**, and higher ML scores in the upper eyelid **(G)**. Compared to complete obstruction, incomplete obstruction showed greater upper eyelid MG loss scores **(A)** but significantly lower OSDI scores **(D)**. *p < 0.05, **p < 0.01, ***p < 0.001.

Notably, obstruction severity emerged as a critical factor. Compared to controls, both incomplete and complete obstruction groups exhibited significantly higher OSDI scores, elevated NITMH, and more severe MG loss (all *p* < 0.001). Crucially, the incomplete obstruction group demonstrated the most severe upper eyelid MG loss among all groups (*p* < 0.001). Pairwise comparisons confirmed that incomplete obstruction was associated with significantly greater upper eyelid MG loss than both controls (*p* < 0.001) and complete obstruction (*p* = 0.002). Eyelid margin and MG orifice scores were significantly worse in both obstruction groups versus controls (all *p* < 0.05), while OSDI scores were highest in complete obstruction (*p* < 0.001 vs. controls and incomplete obstruction). Detailed results of the comparisons across obstruction severity groups are presented in Supplementary Table S4 and illustrated in Figure 2. Dacryocystitis was significantly associated with worse MG loss, orifice, eyelid margin, and Marx line scores (all < 0.05; Supplementary Table S5).

In contrast, disease duration, serum E2 and T levels showed no widespread significant associations with the majority of MG parameters in the univariate analysis (detailed results are presented in Supplementary Tables S6, S7). A pivotal and counterintuitive finding emerged from the analysis of obstruction severity. As summarized in Table 2.

### 3.3 Independent risk factors from multivariable regression analysis

Multivariable regression models were constructed to identify independent risk factors, adjusting for potential confounders. The results of these analyses are presented in Table 3.

**Table 3:** Significant factors associated with ocular surface parameters in postmenopausal women with PANDO.

Dependent variable	Significant predictor	OR/B coefficient	95% CI	*p*	Model performance
Ordered logistic regression models					
Upper eyelid MG loss	Obstruction severity (Incomplete vs. Complete)	OR = 4.68	2.48–8.82	< 0.001	LR χ^2^ = 39.43, *p* < 0.001; Pearson goodness-of-fit *p* = 0.991
MG secretion expressibility	Disease duration (Per year)	OR = 0.96	0.92–0.99	0.027	Nagelkerke R^2^ = 0.088; LR χ^2^ = 14.58, *p* = 0.024; Pearson *p* = 0.399
Lower eyelid meibum quality	Obstruction severity (Incomplete vs. Complete)	OR = 2.02	1.13–3.63	0.019	Nagelkerke R^2^ = 0.087; LR χ^2^ = 14.56, *p* = 0.024; Pearson *p* = 0.284
Linear regression model					
TBUT	NITMH (Per unit)	*B* = 2.85	1.22–4.49	< 0.001	R^2^ = 0.121, adjusted R^2^ = 0.090; Model *F* = 3.91, *p* = 0.001

CI, confidence interval; MG, meibomian gland; NITMH, non-invasive tear meniscus height; OR, odds ratio; PANDO, primary acquired nasolacrimal duct obstruction; TBUT, tear break-up time. Ordered logistic regression models were used for ordinal outcome variables (MG loss, secretion expressibility, meibum quality). Multiple linear regression was used for the continuous outcome (TBUT). OR Interpretation: For obstructive severity, the reference category is Complete obstruction. An OR > 1 indicates higher odds of a more severe condition (e.g., greater MG loss, worse meibum quality) associated with Partial obstruction. For disease duration, the OR represents the change in odds per one-year increase. B Coefficient Interpretation: Represents the change in the dependent variable (TBUT, in seconds) for each one-unit increase in the predictor (NITMH). Model performance metrics were extracted from SPSS outputs: likelihood ratio chi-square (LR χ^2^), Nagelkerke’s pseudo R^2^, Pearson goodness-of-fit test, and for linear regression, R^2^, adjusted R^2^, and the overall F test.

After adjustment for confounders including age and menopause duration, incomplete obstruction remained the strongest independent predictor, conferring a 4.68-fold increased odds of severe upper eyelid MG loss (OR = 4.68, 95% CI: 2.48–8.82, *p* < 0.001) and a 2.02-fold increased odds of worse lower eyelid meibum quality (OR = 2.02, 95% CI: 1.13–3.63, *p* = 0.019). Each additional year of disease duration was associated with slightly reduced odds of severe secretion expressibility impairment (OR = 0.96 per year, 95% CI: 0.92–0.99, *p* = 0.027). A higher NITMH independently predicted a longer TBUT (*B* = 2.85, 95% CI: 1.22–4.49, *p* < 0.001). Notably, several factors significant in the univariate analysis (e.g., age, menopause duration, dacryocystitis) were no longer independently associated with key MG outcome parameters in the multivariable models after adjusting for obstruction completeness.

### 3.4 Exploratory analyses

#### 3.4.1 Sex hormones

In an exploratory analysis of a subgroup with available serum hormone data (*n* = 98), lower testosterone levels were independently associated with worse lower eyelid meibum quality (*B* = −17.120, SE = 6.975, *p* < 0.05). No other consistent associations between systemic hormone levels and MG parameters were observed. Detailed results of these exploratory hormonal analyses are presented in Supplementary Table S8.

#### 3.4.2 Tear cytokines

To probe the inflammatory basis, tear cytokine analysis was performed in a consecutively recruited subset (*n* = 71). This subset comprised: (1) a control group (*n* = 24) of age-matched postmenopausal women without PANDO, significant dry eye, or MGD; (2) patients with incomplete obstruction (*n* = 23); and (3) patients with complete obstruction (*n* = 24). The three groups were comparable, with no significant differences in age or menopause duration (all *p* > 0.05; Table 4).

**Table 4:** Cytokine and clinical variable levels across different obstruction degrees.

Parameter	Control group (*N* = 24)	Incomplete obstruction group (*N* = 23)	Complete obstruction group (*N* = 24)	*p*
Age (years)	59.00 [56.50, 65.50]	59.50 [55.50, 70.00]	58.00 [56.00, 69.00]	0.860
Menopause duration (years)	9.00 [4.50, 14.00]	4.00 [2.00, 9.50]	9.00 [5.00, 18.00]	0.278
Disease duration (years)	-	4.50 [1, 7]	5 [0.75, 10]	0.912
IL-6 (pg/mL)	3.50 [2.81, 5.26]	4.38 [2.51, 13.89]	9.56 [2.32, 19.82]	0.017*
IL-8 (pg/mL)	117.30 [74.98, 197.53]	236.19 [63.38, 411.52]	179.45 [84.87, 493.17]	0.049*
TNF-α (pg/mL)	0.41 [0.11, 0.74]	0.74 [0.44, 2.21]	0.74 [0.20, 2.35]	0.076
IL-1α (pg/mL)	3.63 [2.15, 4.44]	3.34 [1.82, 15.57]	4.69 [3.32, 5.99]	0.092
IL-1β (pg/mL)	3.13 [1.54, 3.65]	3.39 [1.71, 7.61]	4.56 [2.62, 7.61]	0.247
MIP-1α (pg/mL)	198.43 [170.93, 214.16]	200.38 [136.78, 290.45]	193.08 [132.17, 332.35]	0.170
IL-10 (pg/mL)	2.87 [2.73, 4.26]	2.46 [1.79, 3.39]	1.91 [1.18, 2.61]	0.072

Data are presented as [*M* (P25, P75)]. IL, interleukin; MIP, macrophage inflammatory protein, TNF, tumor necrosis factor. Kruskal–Wallis H test and Holm–Bonferroni method were used for cytokine level comparison. *Statistical significance was defined as *p* < 0.05.

The concentrations of IL-6 and IL-8 differed significantly across the three groups (*p* = 0.017 and *p* = 0.049, respectively, Kruskal-Wallis H test). Post-hoc analyses showed significantly elevated IL-6 (*p* = 0.014) and IL-8 (*p* = 0.049) levels in the complete obstruction group compared to controls. Notably, the incomplete obstruction group exhibited intermediate levels of both IL-6 and IL-8, which were not statistically different from either the control or the complete obstruction groups. In contrast, no significant differences were observed in the levels of TNF-*α*, IL-10, IL-1α, IL-1β, and MIP-1α among the three groups (all *p* > 0.05; Table 4).

### 3.5 Meibomian gland morphological features

**Figure 3:**
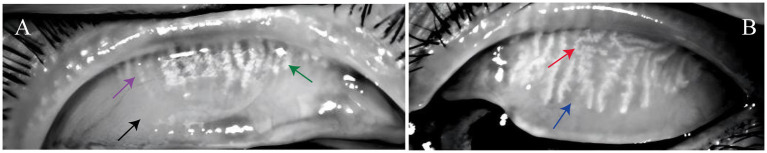
Meibomian gland morphological features in postmenopausal women with PANDO. **(A)** Extensive gland loss (black arrow), glandular dilation (green arrow), and glandular atrophy and thinning (purple arrow). **(B)** Gland tortuosity and hook-shaped deformation (red arrow), interglandular space widening (blue arrow).

As illustrated in Figure 3, postmenopausal women with PANDO exhibited various structural abnormalities, including, but not limited to, gland tortuosity, interglandular space widening, glandular atrophy, glandular dilation, gland discontinuity, hook-shaped deformation, and extensive gland loss.

## 4 Discussion

### 4.1 The principal clinical finding and its implications

The most salient finding of our study is the identification of incomplete rather than complete NLD obstruction as the predominant factor independently associated with severe MG loss and dysfunction in postmenopausal women with PANDO. The magnitude of this association (OR = 4.68 for upper eyelid MG loss) underscores a profound and previously underappreciated clinical risk. This compels a significant shift in clinical vigilance. Contrary to conventional paradigms, the patient presenting with mild, intermittent epiphora from a partially blocked lacrimal system is at a substantially greater risk for silent, irreversible MG atrophy than the patient with profound, constant tearing from a complete obstruction. This finding fundamentally redefines risk stratification in PANDO and argues for the integration of meibography, particularly of the upper eyelids, as a crucial diagnostic tool in all PANDO patients, regardless of symptom severity. Extending beyond our previous descriptive findings that established a high prevalence of MGD in this population (6, 27, 28), the present study identifies—for the first time—that incomplete obstruction, rather than complete obstruction, is the principal independent factor associated with severe MGD.

### 4.2 Interpretation of univariate and multivariable findings

The addition of a control group allowed us to calibrate the absolute burden of PANDO on the ocular surface. Our univariate analysis initially identified several factors, including advanced age, prolonged menopause duration, and the presence of dacryocystitis, that were associated with adverse MG parameters, which is consistent with the known pathophysiology of MGD (16–18, 29–31). Notably, the control group consistently displayed the least pathological changes, validating the study design. However, the critical insight from our study emerged from the multivariable regression models, which accounted for potential confounding and interactions between these variables. In these adjusted models, the strong association between incomplete obstruction and severe MG damage persisted with remarkable potency, whereas the effects of age, menopause duration, and dacryocystitis were attenuated and lost independent statistical significance. This pivotal finding indicates that the influence of these demographic and inflammatory factors, while present, is likely secondary to, or subsumed within, the dominant pathophysiological pathway initiated by the dysfunctional drainage characteristic of incomplete obstruction. It appears that the local ocular surface environment dictated by the completeness of obstruction overrides the contributions of these more systemic or concomitant factors in association with severe glandular damage.

Building on our multivariable findings which identified lower testosterone as an independent risk factor for meibum quality, our exploratory analysis of sex hormones offers a nuanced perspective to the dominant inflammatory narrative. While systemic levels showed no broad associations, the isolated link between lower testosterone and worse meibum quality provides a biological clue that cannot be dismissed. This suggests that in the context of PANDO, the systemic endocrine milieu (e.g., postmenopausal androgen decline) may act as a “disease modifier” rather than a primary driver (16, 28). It could potentially lower the repair threshold of the glands to inflammatory damage or alter lipid composition, thereby acting synergistically with local inflammation to exacerbate dysfunction. This concept of a synergistic systemic susceptibility offers a deeper, supra-anatomical rationale for why postmenopausal women represent a demographic at high risk for both PANDO and MGD.

### 4.3 Toward a new pathophysiological model: integrating clinical and exploratory data

**Figure 4:**
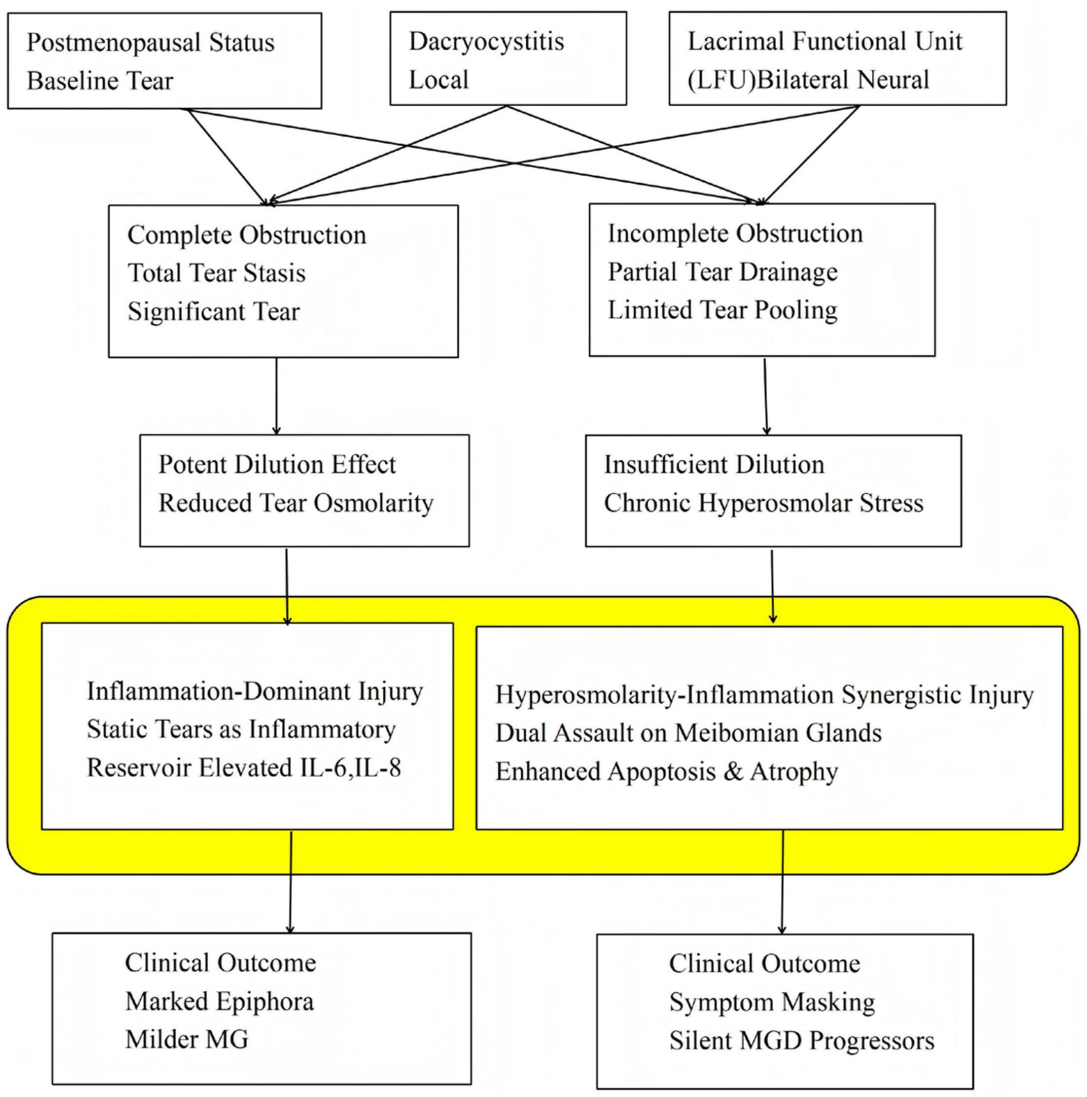
Proposed pathophysiological model: “Hyperosmolarity-inflammation synergistic injury” in postmenopausal women with PANDO.

The inclusion of a control group provides a normative benchmark for tear dynamics and inflammatory mediators. Our initial hypothesis was that luminal narrowing in incomplete NLD might intensify inflammation. However, our exploratory cytokine data, which showed the highest inflammatory markers in the complete obstruction group, do not support a model in which inflammation magnitude alone drives the observed glandular damage. Instead, we propose a “Hyperosmolarity-Inflammation Synergistic Injury” model (Figure 4), which integrates established concepts of tear film homeostasis with our clinical findings.

Tear film osmolarity is a central driver of ocular surface inflammation and damage (32). In healthy individuals, tear osmolarity is approximately 302 ± 9.7 mOsm/L, rising to 315–336 mOsm/L in dry eye. Mathematical modeling and fluorescence quenching studies indicate that within tear film breakup areas, local osmolarity can spike to 1900–3,000 mOsm/L – far exceeding meniscus values. These “hyperosmolarity hotspots” are directly cytotoxic to meibomian gland acinar cells, promoting apoptosis and inflammatory cytokine release.

The ocular surface of postmenopausal women rests on a fragile physiological foundation. Early evidence suggested that this population exhibits inherent ocular surface susceptibility, as even asymptomatic postmenopausal women show tear osmolarity at the upper limit of the normal range (33). More recently, the large-scale DREAM study confirmed that postmenopausal women have significantly higher baseline tear osmolarity than premenopausal women (34). Within this context, the nature of nasolacrimal duct obstruction may exert divergent effects.

Complete obstruction leads to marked tear retention and a pronounced lacrimal lake. This excess volume dilutes tear solutes, mitigating baseline hyperosmolarity and shifting the ocular surface toward an iso-osmolar state. This “osmotic rescue” is supported by several studies: Yuksel et al. found significantly lower preoperative tear osmolarity in PANDO eyes than in healthy controls (35); Stahl et al. reported normal tear osmolarity despite markedly elevated tear meniscus height (36). Moreover, Saleh et al. directly demonstrated that tear osmolarity is significantly lower in patients with epiphora than in controls (37). Although theoretically protective, static tear stasis fosters high-grade inflammation, as evidenced by our elevated IL-6 and IL-8 levels. Thus, the injury mechanism in complete obstruction can be understood as inflammation-dominant.

Incomplete obstruction presents a more detrimental scenario. The limited increase in tear volume insufficiently corrects the baseline hyperosmolarity of postmenopausal women, leading to chronic, unmitigated hyperosmolar stress. This stress is directly cytotoxic to meibomian gland epithelial cells, promoting apoptosis and gland dropout (18, 19). Concurrently, impaired drainage allows accumulation of inflammatory mediators (38, 39). The ocular surface therefore suffers a dual, synergistic assault from persistent hyperosmolarity and inflammation.

This local synergistic injury may be further amplified by neural mechanisms involving the lacrimal functional unit (LFU). The LFU comprises the ocular surface, lacrimal glands, and their interconnecting innervation (40). Afferent signals from the cornea travel via the trigeminal nerve to the superior salivatory nucleus; efferent parasympathetic fibers project through the pterygopalatine ganglion to the lacrimal and meibomian glands, regulating secretion (41). Cold thermoreceptors on the ocular surface detect changes in tear film temperature and osmolarity, reflexly maintaining basal tear production and blinking rate (42). Secretion of the main lacrimal gland is regulated dominantly by autonomic parasympathetic nerves, reflexly activated by eye surface sensory nerves (40). Sensory nerve endings release neuropeptides (substance P, CGRP), inducing neurogenic inflammation that links neural activity to ocular surface damage (32, 43).

Xiao et al. showed that tear duct obstruction in mice causes lacrimal gland dysfunction and altered secretory vesicle proteins (Rab3d, Vamp8, Snap23), supporting a “nose-tear duct-superior salivatory nucleus-lacrimal gland axis” (1). This pathway may be particularly relevant in incomplete obstruction: persistent abnormal afferent signals from the incompletely obstructed passage could dysregulate reflex regulation, amplifying hyperosmolar-inflammatory damage to meibomian glands. Thus, this phenotype reflects a “Hyperosmolarity-Inflammation Synergistic” injury, integrating tear osmolarity dynamics, local inflammation, and LFU neural regulation.

### 4.4 Dynamic compensation and symptom dissociation

Our findings reveal two parallel compensatory mechanisms that decouple symptoms from structural damage. First, within the MGs, residual acini upregulate secretory function to compensate for early dropout, evidenced by the inverse association between disease duration and expressibility (OR = 0.96/year). This compensatory hyperplasia, supported by unipotent progenitor cells (44), temporarily preserves lipid layer integrity (45). Second, in the lacrimal system, pathological tear retention creates an aqueous reservoir (elevated NITMH predicting longer TBUT) that mechanically stabilizes the tear film. Together, these processes create “silent MGD progressors”---patients with significant gland loss but minimal symptoms, a phenomenon echoed in bilateral tear instability patterns (46). This intricate compensation, occurring alongside chronic inflammation (3, 47), mandates a diagnostic shift beyond symptoms to integrated meibography and lacrimal function assessment (45).

### 4.5 Clinical implications: from risk stratification to precision management

These findings inform a more nuanced clinical approach. Risk Stratification: Older, long-postmenopausal women with incomplete PANDO, particularly those with dacryocystitis, represent a profile meriting heightened vigilance for occult MGD. Diagnostic Recommendation: Given the documented symptom-structure dissociation, our data support integrating meibography (especially of the upper eyelids) into the standard PANDO workup to detect silent disease progression. Therapeutic Considerations: The treatment objective may reasonably be expanded beyond resolving epiphora to encompass ocular surface preservation. For patients with high-risk features, early intervention (e.g., dacryocystorhinostomy) can be considered, as evidence suggests it may ameliorate the underlying tear film dysfunction by restoring physiological drainage (35, 46). Concomitant MGD management should be part of the comprehensive treatment strategy.

### 4.6 Study limitations and future directions

This study has several limitations that warrant consideration and provide directions for future research. First, the cross-sectional design identifies significant associations but cannot establish causality. Nevertheless, it provides robust preliminary evidence for the novel hypothesis that incomplete obstruction poses a greater risk for severe MG damage, forming a critical foundation for longitudinal studies to confirm this temporal relationship. Second, while conducted at a single center, all examinations followed stringent, standardized protocols in a well-defined clinical population, ensuring strong internal validity; the primary conclusion thus retains significant clinical import. Third, the hormonal subgroup was relatively modest in size (*n* = 98), limiting the statistical power for a comprehensive multivariable analysis of hormonal effects. Furthermore, systemic hormone levels may not precisely reflect local glandular hormonal activity. Despite this, our exploratory analysis yielded the salient finding linking lower testosterone to worse meibum quality, offering a valuable clue regarding the potential role of systemic endocrine status as a disease modifier. Fourth, although we assessed a panel of key inflammatory cytokines, the tear inflammatory network is complex, and other unmeasured mediators may be involved. Finally, our proposed “Hyperosmolarity-Inflammation Synergistic Injury” model, while physiologically plausible and consistent with our clinical parameter patterns and supportive external literature (35, 46), lacks direct validation through contemporaneous tear osmolarity measurements. This does not diminish the importance of our core clinical finding but rather highlights a crucial target for confirmation in future investigations.

Critically, these methodological considerations do not undermine the robustness or clinical significance of our primary conclusion. Through rigorous multivariable analysis, this study is the first to identify incomplete—not complete—NLD obstruction as the predominant independent risk factor for severe MG loss and dysfunction in postmenopausal women with PANDO. This counterintuitive finding, based on objective meibography and adequately adjusted statistics, challenges prevailing clinical paradigms and carries immediate, transformative implications for risk stratification and management in PANDO. Future studies employing longitudinal designs, multi-center collaboration, integration of local hormone assessment and omics technologies, and direct measurement of tear osmolarity will be essential to validate and extend the pathophysiological model proposed here, ultimately advancing toward precision management of this complex condition.

## 5 Conclusion

This study establishes a critical, revised risk paradigm for a common clinical condition: incomplete—not complete—NLD obstruction is the predominant factor independently associated with MGD in postmenopausal women with PANDO in this cross-sectional study. This finding directly challenges the conventional association between symptom severity and disease burden, identifying patients with mild, intermittent epiphora as being at greatest risk for occult, structural damage. Therefore, clinical practice must evolve. Assessment of obstruction completeness is essential for risk stratification. We recommend integrating meibography, particularly of the upper eyelids, into the standard diagnostic evaluation for postmenopausal women with PANDO, irrespective of symptomatic presentation. This approach is vital to detect silent disease progression and to guide management strategies that address not only epiphora but also the preservation of MG health.

## Data Availability

The raw data supporting the conclusions of this article will be made available by the authors, without undue reservation.
