# Supplementary material for: Incomplete rather than complete nasolacrimal duct obstruction Is strongly associated with meibomian gland dysfunction in postmenopausal women with PANDO: a cross-sectional study
Source: Front Med (Lausanne). 2026 Apr 30;13:1831157. doi: 10.3389/fmed.2026.1831157 (PMC13171326; doi:10.3389/fmed.2026.1831157)
Supplement: Supplementary file 1 [file Table_1.DOCX]

**Table 1 Structural and Functional Analysis of Meibomian Glands Across Age Groups**

|  | **Age <60 years**  （N=100） | **Age ≥60**  （N=80 ） | **Z value** | **P** |
| --- | --- | --- | --- | --- |
| **Upper eyelid MG loss** (score) | 1[1 ，2] | 2[1 ，3] | 2.418 | 0.016 |
| **Lower eyelid MG loss** (score) | 1[1 ，1] | 1[1 ，2] | 3.657 | <0.001 |
| MG orifices (score) | 2[1 ，2] | 2[2 ，2] | 2.824 | 0.005 |
| MG secretion expressibility (score) | 2[1 ，2] | 2[2 ，2] | 1.418 | 0.156 |
| **Upper eyelid** meibum quality (score) | 1[1 ，2] | 1[1 ，3] | 0.518 | 0.604 |
| **Lower eyelid** meibum quality (score) | 1[1 ，2] | 1[1 ，2] | -0.326 | 0.744 |
| eyelid margins (score) | 2[2 ，3] | 3[2 ，4] | 3.606 | <0.001 |
| Upper eyelid ML (score) | 4[2 ，6] | 6[4 ，7.75] | 3.717 | <0.001 |
| Lower eyelid ML (score) | 6[4 ，6.75] | 6[4 ，7] | 0.427 | 0.669 |
| TBUT | 3[2 ，5] | 2[2 ，4] | -2.088 | 0.037 |
| CFS | 1[0 ，2] | 1[0 ，1] | 0.267 | 0.790 |
| OSDI (score) | 33.33[17.86 ，52.78] | 33.33[19.44 ，54.95] | 0.451 | 0.652 |
| NITMH (mm) | 0.42[0.30, 0.60] | 0.40[0.25, 0.60] | 0.668 | 0.504 |

MG: meibomian gland; ML: Marx's line; TBUT: tear film breakup time; CFS: corneal fluorescein staining; OSDI: ocular surface disease index;

The Mann-Whitney U test was applied for comparisons among age groups.

Statistical significance was defined as P < 0.05. P > 0.05 . *P < 0.05, **P < 0.01, ***P < 0.001.

N = 180 refers to patients with PANDO only; healthy controls are not included in this table.
