# Supplementary material for: Incomplete rather than complete nasolacrimal duct obstruction Is strongly associated with meibomian gland dysfunction in postmenopausal women with PANDO: a cross-sectional study
Source: Front Med (Lausanne). 2026 Apr 30;13:1831157. doi: 10.3389/fmed.2026.1831157 (PMC13171326; doi:10.3389/fmed.2026.1831157)
Supplement: Supplementary file 2 [file Table_2.DOCX]

**Table 2 Structural and Functional Analysis of Meibomian Glands Across Menopause duration Groups**

|  | **MD < 10 years**  （N=100） | **MD ≥10 years**  （N=80 ） | **Z value** | **P** |
| --- | --- | --- | --- | --- |
| **Upper eyelid MG loss** (score) | 1[1 ，2] | 2[1 ，3] | 3.601 | <0.001 |
| **Lower eyelid MG loss** (score) | 1[1 ，1] | 1[1 ，2] | 4.662 | <0.001 |
| MG orifices (score) | 2[1 ，2] | 2[2 ，2] | 1.930 | 0.05 |
| MG secretion expressibility (score) | 2[1 ，2] | 2[2 ，3] | 3.161 | 0.002 |
| **Upper eyelid** meibum quality (score) | 2[1 ，2] | 1[1 ，3] | 0.092 | 0.926 |
| **Lower eyelid** meibum quality (score) | 1[1 ，2] | 1[1 ，2] | -0.503 | 0.615 |
| eyelid margins (score) | 3[2 ，3.75] | 3[2 ，4] | 2.821 | 0.005 |
| Upper eyelid ML (score) | 4[2 ，6] | 6[4 ，8] | 3.698 | <0.001 |
| Lower eyelid ML (score) | 6[4 ，7] | 6[4 ，7] | 0.199 | 0.843 |
| TBUT | 3[2 ，5] | 2[1.25 ，4] | -2.469 | 0.014 |
| CFS | 1[0 ，2] | 1[0 ，1] | 1.428 | 0.153 |
| OSDI (score) | 33.33[16.97 ，50.00] | 33.33[19.83 ，56.08] | 0.765 | 0.445 |
| NITMH (mm) | 0.42[0.32, 0.60] | 0.39[0.23, 0.60] | -1.263 | 0.207 |

MD: menopause duration; MG: meibomian gland; ML: Marx's line; TBUT: tear film breakup time; CFS: corneal fluorescein staining; OSDI: ocular surface disease index; NITMH: non-invasive tear meniscus height ;The Mann-Whitney U test was applied for comparisons among menopause duration groups. Statistical significance was defined as P < 0.05. P > 0.05 . *P < 0.05, **P < 0.01, ***P < 0.001.

N = 180 refers to patients with PANDO only; healthy controls are not included in this table.
