# Supplementary material for: Incomplete rather than complete nasolacrimal duct obstruction Is strongly associated with meibomian gland dysfunction in postmenopausal women with PANDO: a cross-sectional study
Source: Front Med (Lausanne). 2026 Apr 30;13:1831157. doi: 10.3389/fmed.2026.1831157 (PMC13171326; doi:10.3389/fmed.2026.1831157)
Supplement: Supplementary file 3 [file Table_3.DOCX]

**Table 3 Structural and Functional Analysis of Meibomian Glands Across Disease Duration Groups**

|  | disease duration ≤ 1year  ( N=54) | 1< disease duration ≤ 5 years  (N=63) | disease duration>5 years  (N=63) | *H value* | P |
| --- | --- | --- | --- | --- | --- |
| **Upper eyelid MG loss** (score) | 2[1 ，2] | 1[1 ，2] | 2[1 ，2] | 1.187 | 0.552 |
| **Lower eyelid MG loss** (score) | 1[1 ，2] | 1[1 ，2] | 1[1 ，2] | 1.092 | 0.579 |
| MG orifices (score) | 2[1 ，2] | 2[2 ，2] | 2[1 ，2] | 2.717 | 0.257 |
| MG secretion expressibility (score) | 2[2 ，2] | 2[1 ，3] | 2[1 ，2] | 2.605 | 0.272 |
| **Upper eyelid** meibum quality (score) | 1[1 ，3] | 2[1 ，3] | 1[1 ，2] | 1.053 | 0.591 |
| **Lower eyelid** meibum quality (score) | 1[1 ，3] | 1[1 ，2] | 1[1 ，2] | 3.603 | 0.165 |
| eyelid margins (score) | 3[2 ，4] | 3[2 ，4] | 3[2 ，4] | 3.492 | 0.174 |
| Upper eyelid ML (score) | 6[3 ，7] | 5[3 ，7] | 5[3 ，6] | 2.806 | 0.246 |
| Lower eyelid ML (score) | 6[4 ，7] | 6[4 ，7] | 6[4 ，7] | 0.900 | 0.638 |
| TBUT | 2[1 ，4] | 3[1 ，5] | 3[2 ，5] | 5.668 | 0.059 |
| CFS | 1[0 ，2] | 1[0 ，1] | 1[0 ，1] | 1.491 | 0.474 |
| OSDI (score) | 33.33[17.86，53.74] | 31.25[18.75，53. 13] | 33.33[17.86，52.78] | 0.222 | 0.895 |
| NITMH (mm) | 0.39[0.27, 0.57] | 0.41[0.29, 0.58] | 0.46[0.31, 0.64] | 1.983 | 0.371 |

MG: meibomian gland; ML: Marx's line; TBUT: tear film breakup time; CFS: corneal fluorescein staining; OSDI: ocular surface disease index; NITMH: non-invasive tear meniscus height ;The Kruskal-Wallis H test was applied for comparisons among disease duration groups.

Statistical significance was defined as P < 0.05. P > 0.05 . *P < 0.05, **P < 0.01, ***P < 0.001.

N = 180 refers to patients with PANDO only; healthy controls are not included in this table.
