# Supplementary material for: Incomplete rather than complete nasolacrimal duct obstruction Is strongly associated with meibomian gland dysfunction in postmenopausal women with PANDO: a cross-sectional study
Source: Front Med (Lausanne). 2026 Apr 30;13:1831157. doi: 10.3389/fmed.2026.1831157 (PMC13171326; doi:10.3389/fmed.2026.1831157)
Supplement: Supplementary file 4 [file Table_4.DOCX]

**Table 4 Structural and Functional Analysis of Meibomian Glands Across Obstruction Severity Groups**

|  | Control group  ( N=92 ) | **Incomplete obstruction**  ( N=75 ) | **Complete obstruction**  ( N=75 ) | *H value* | *P* |
| --- | --- | --- | --- | --- | --- |
| **Upper eyelid MG loss** (score) | 1[1 , 2] | 2[1, 2]^a*** | 1[1 , 2]^b*** | 16.228 | <0.001 |
| **Lower eyelid MG loss** (score) | 1[1 , 1] | 1[1, 2]^a* | 1[1, 1]^a* | 7.391 | 0.025 |
| MG orifices (score) | 2[0, 2] | 2[1.75, 2]^a* | 2[1 , 2] | 6.808 | 0.033 |
| MG secretion expressibility (score) | 2[1, 2] | 2[1, 2] | 2[1, 2] | 1.227 | 0.542 |
| **Upper eyelid** meibum quality (score) | 1[1, 2] | 1.5[1, 3] | 1[1, 2] | 1.573 | 0.455 |
| **Lower eyelid** meibum quality (score) | 1[1 , 2] | 1[1, 3] | 1[1, 2] | 4.608 | 0.100 |
| eyelid margins (score) | 2[1, 3] | 3[2 , 4]^a*** | 3[2, 4]^a*** | 22.976 | <0.001 |
| Upper eyelid ML (score) | 2[1, 4.75] | 5[3, 6]^a*** | 5[3, 7]^a*** | 31.169 | <0.001 |
| Lower eyelid ML (score) | 5[2, 7] | 6[4, 7] | 6[4, 7] | 5.888 | 0.053 |
| TBUT | 3[2, 5] | 3[2 , 4] | 3[2, 5] | 0.532 | 0.767 |
| CFS | 1[0, 2] | 1[0 , 1.25] | 1[0 , 2] | 0.156 | 0.925 |
| OSDI (score) | 10.57[2.78 , 22.22] | 25.00[12.50,44.67]^a*** | 40.63[25.00, 57.5]^a***,b** | 62.215 | <0.001 |
| NITMH (mm) | 0.22[0.18, 0.27] | 0.39[0.27, 0.50]^a*** | 0.49[0.3, 0.67]^a*** | 82.247 | <0.001 |

Data are presented as median [interquartile range].This analysis was performed on 150 patients with PANDO (75 with incomplete obstruction, 75 with complete obstruction) after age- and menopause duration-matching. The remaining 30 patients were excluded as they could not be adequately matched.

MG, meibomian gland; ML, Marx's line; TBUT, tear film breakup time; CFS, corneal fluorescein staining; OSDI, Ocular Surface Disease Index; NITMH, non-invasive tear meniscus height.

Kruskal–Wallis test was used for group comparisons. Pairwise comparisons were performed with Bonferroni correction. Statistical significance was defined as

P < 0.05. P > 0.05 . *P < 0.05, **P < 0.01, ***P < 0.001.

^a Significantly different from control group.

^b Significantly different from incomplete obstruction group.
