# Supplementary material for: Incomplete rather than complete nasolacrimal duct obstruction Is strongly associated with meibomian gland dysfunction in postmenopausal women with PANDO: a cross-sectional study
Source: Front Med (Lausanne). 2026 Apr 30;13:1831157. doi: 10.3389/fmed.2026.1831157 (PMC13171326; doi:10.3389/fmed.2026.1831157)
Supplement: Supplementary file 5 [file Table_5.DOCX]

**Table 5 Structural and Functional Analysis of Meibomian Glands Across **Dacryocystitis** Groups**

|  | Control group  ( N=92 ) | **Without dacryocystitis**  ( N=72 ) | **With dacryocystitis**  ( N=72 ) | **H value** | *P* |
| --- | --- | --- | --- | --- | --- |
| **Upper eyelid MG loss** (score) | 1[1, 2] | 1[1, 2]^a* | 2[1, 2] | 7.816 | 0.020 |
| **Lower eyelid MG loss** (score) | 1[1, 1] | 1[1, 1] | 1[1, 1] | 4.364 | 0.113 |
| MG orifices (score) | 2[0 , 2] | 2[1, 2] | 2[1 , 2]^a* | 7.325 | 0.026 |
| MG secretion expressibility (score) | 2[1, 2] | 2[1, 2] | 2[1, 3] | 1.042 | 0.594 |
| **Upper eyelid** meibum quality (score) | 1[1, 2] | 1.5[1, 3] | 2[1, 3] | 1.725 | 0.422 |
| **Lower eyelid** meibum quality (score) | 1[1, 2] | 1[1 , 2] | 2[1, 2.75] | 2.602 | 0.272 |
| eyelid margins (score) | 2[1, 3] | 2[2, 3] | 3[2 , 4]^a*** | 21.825 | <0.001 |
| Upper eyelid ML (score) | 2[1, 4.75] | 6[3, 6]^a*** | 5[2.25, 6]^a*** | 28.466 | <0.001 |
| Lower eyelid ML (score) | 5[2 , 7] | 6[5, 7] | 6[5, 7] | 7.181 | 0.028 |
| TBUT | 3[2, 5] | 3[2 , 4] | 3[1.25, 5] | 0.074 | 0.963 |
| CFS | 1[0, 2] | 1[0, 2] | 1[0 , 1] | 1.245 | 0.537 |
| OSDI (score) | 10.57[2.78, 22.22] | 28.57[16.97 ,50.00]^a*** | 39.09[19.94, 55.56]^a*** | 58.009 | <0.001 |
| NITMH (mm) | 0.22[0.18, 0.27] | 0.40[0.27, 0.64]^a*** | 0.45[0.30, 0.58]^a*** | 78.059 | <0.001 |

Data are presented as median [interquartile range]. This analysis was performed on 144 patients with PANDO (72 with dacryocystitis, 72 without) after age- and menopause duration-matching. The remaining 36 patients were excluded because they could not be adequately matched. Healthy controls (n = 92) are shown for reference.

MG, meibomian gland; ML, Marx's line; TBUT, tear film breakup time; CFS, corneal fluorescein staining; OSDI, Ocular Surface Disease Index; NITMH, non-invasive tear meniscus height.

Kruskal–Wallis test was used for group comparisons. Pairwise comparisons were performed with Bonferroni correction. Statistical significance was defined as P < 0.05. P > 0.05 . *P < 0.05, **P < 0.01, ***P < 0.001.

^a Significantly different from control group.
