# Supplementary material for: Incomplete rather than complete nasolacrimal duct obstruction Is strongly associated with meibomian gland dysfunction in postmenopausal women with PANDO: a cross-sectional study
Source: Front Med (Lausanne). 2026 Apr 30;13:1831157. doi: 10.3389/fmed.2026.1831157 (PMC13171326; doi:10.3389/fmed.2026.1831157)
Supplement: Supplementary file 6 [file Table_6.DOCX]

**Table 6 Structural and Functional Analysis of Meibomian Glands Across Estradiol Groups**

|  | Estradiol <10pg/ml  ( N=31 ) | Estradiol ≥10pg/ml  ( N=31 ) | Z value | P |
| --- | --- | --- | --- | --- |
| **Upper eyelid MG loss** (score) | 2[1 ，2] | 2[1 ，2] | 5.12 | 0.609 |
| **Lower eyelid MG loss** (score) | 1[1 ，1] | 1[1 ，1] | -0.486 | 0.627 |
| MG orifices (score) | 2[1 ，2] | 2[1 ，3] | 0.319 | 0.75 |
| MG secretion expressibility (score) | 2[1 ，3] | 2[2 ，2] | -0.359 | 0.719 |
| **Upper eyelid** meibum quality (score) | 1[1 ，3] | 1[1 ，2] | -0.986 | 0.324 |
| **Lower eyelid** meibum quality (score) | 2[1 ，2] | 1[1 ，3] | 1.329 | 0.184 |
| eyelid margins (score) | 3[2 ，3] | 2[1 ，3] | -1.481 | 0.139 |
| Upper eyelid ML (score) | 5[2 ，6] | 4[1 ，6] | -0.796 | 0.426 |
| Lower eyelid ML (score) | 6[4 ，7] | 6[6 ，7] | -1.902 | 0.057 |
| TBUT | 3[2 ，5] | 3[2 ，5] | -0.301 | 0.763 |
| CFS | 1[0 ，2] | 1[0 ，1] | 0.215 | 0.83 |
| OSDI (score) | 33.33[18.75 ，53.57] | 33.33[17.86 ，53. 13] | -0.056 | 0.955 |
| NITMH (mm) | 0.34[0.29, 0.64] | 0.48[0.34, 0.84] | 1.493 | 0.135 |

Data are presented as median [interquartile range]. n=62 patients with valid estradiol data. MG: meibomian gland; ML: Marx's line; TBUT: tear film breakup time; CFS: corneal fluorescein staining; OSDI: ocular surface disease index; NITMH: non-invasive tear meniscus height ;The Mann-Whitney U test was applied for comparisons among estradiol groups. Statistical significance was defined as P < 0.05.

P > 0.05 . *P < 0.05, **P < 0.01, ***P < 0.001.
