# Supplementary material for: Incomplete rather than complete nasolacrimal duct obstruction Is strongly associated with meibomian gland dysfunction in postmenopausal women with PANDO: a cross-sectional study
Source: Front Med (Lausanne). 2026 Apr 30;13:1831157. doi: 10.3389/fmed.2026.1831157 (PMC13171326; doi:10.3389/fmed.2026.1831157)
Supplement: Supplementary file 7 [file Table_7.DOCX]

**Table 7 Structural and Functional Analysis of Meibomian Glands Across Testosterone Groups**

|  | T ≤ 0.8nmol/L  ( N=47 ) | T > 0.8nmol/L  ( N=51) | *Z value* | P |
| --- | --- | --- | --- | --- |
| **Upper eyelid MG loss** (score) | 2[1 ，2] | 2[1 ，2] | 0.733 | 0.464 |
| **Lower eyelid MG loss** (score) | 1[1 ，1] | 1[1 ，2] | 0.07 | 0.944 |
| MG orifices (score) | 2[1 ，2] | 2[1 ，2] | 0 | 1.00 |
| MG secretion expressibility (score) | 2[1 ，2] | 2[2 ，2] | 0.388 | 0.698 |
| **Upper eyelid** meibum quality (score) | 2[1 ，3] | 2[1 ，2] | 1.255 | 0.209 |
| **Lower eyelid** meibum quality (score) | 2[1 ，2] | 1.5[1 ，3] | 0.156 | 0.876 |
| eyelid margins (score) | 3[2 ，3] | 3[2 ，4] | 0.559 | 0.576 |
| Upper eyelid ML (score) | 4[2 ，6] | 6[3 ，7] | 1.255 | 0.209 |
| Lower eyelid ML (score) | 6[4 ，7] | 6[5.75 ，7] | 0.443 | 0.658 |
| TBUT | 3[2 ，4] | 3[2 ，5] | 0.975 | 0.329 |
| CFS | 1[0 ，1] | 1[0 ，2] | 0.233 | 0.815 |
| OSDI (score) | 33.33[20.83 ，55.56] | 33.33[16.67 ，52.78] | -0.612 | 0.541 |
| NITMH (mm) | 0.41[0.27, 0.68] | 0.46[0.32, 0.63] | 0.736 | 0.462 |

T: testosterone; MG: meibomian gland; ML: Marx's line; TBUT: tear film breakup time; CFS: corneal fluorescein staining; OSDI: ocular surface disease index; NITMH: non-invasive tear meniscus height ;The Mann-Whitney U test was applied for comparisons among testosterone groups. Statistical significance was defined as P < 0.05. P > 0.05 . *P < 0.05, **P < 0.01, ***P < 0.001.
