# Supplementary material for: Incomplete rather than complete nasolacrimal duct obstruction Is strongly associated with meibomian gland dysfunction in postmenopausal women with PANDO: a cross-sectional study
Source: Front Med (Lausanne). 2026 Apr 30;13:1831157. doi: 10.3389/fmed.2026.1831157 (PMC13171326; doi:10.3389/fmed.2026.1831157)
Supplement: Supplementary file 8 [file Table_8.DOCX]

| **Parameter** | **Lower Eyelid Meibum Quality Score**  **(Ordinal)** | **CFS**  **(Ordinal)** | **TBUT (Linear)** |
| --- | --- | --- | --- |
| ****Sex Hormones**** |  |  |  |
| E2 (Estradiol) | 0.101 (0.052)† | 0.018 (0.020) | 0.022 (0.021) |
| **Testosterone** | ****-17.120 (6.975)***** | 3.424 (2.070)† | 2.668 (2.082) |
| ****Clinical Factors**** |  |  |  |
| Age | 1.280 (0.704)† | 0.140 (0.155) | -0.187 (0.174) |
| Menopause duration | -1.536 (0.896)† | 0.044 (0.204) | 0.172 (0.218) |
| Disease duration | -0.816 (0.530) | -0.172 (0.226) | 0.256 (0.190) |
| Obstruction severity | -10.204 (6.351) | 1.243 (1.609) | -0.703 (1.655) |
| Dacryocystitis | 2.536 (2.003) | 0.702 (1.143) | 0.071 (1.218) |
| ****NITMH**** | 7.714 (5.589) | ****-11.865 (4.688)***** | 1.449 (2.057) |
| ****Model Statistics**** |  |  |  |
| Fit statistic | χ² = 28.815 | χ² = 19.619 | F = 1.424 |
| P-value | ****0.002**** | 0.051⁽ᵐ⁾ | 0.276 |
| R² | 0.714 (Cox-Snell) | 0.574 (Cox-Snell) | 0.169 (Adj. R²) |

### ****Table S1. Associations of Sex Hormones and Clinical Factors with Ocular Surface Parameters in Postmenopausal Women with PANDO: Multivariate Regression Analysis****

Data are presented as regression coefficient (standard error). *P < 0.05, †P < 0.1 (marginally significant for predictors)
CFS, Corneal Fluorescein Staining; TBUT, Tear Film Breakup Time; E2, Estradiol; NITMH, Non-Invasive Tear Meniscus Height; PANDO, Primary Acquired Nasolacrimal Duct Obstruction
Ordinal regression coefficients represent the log-odds of being in a higher category per unit increase in the predictor.
****¹ Models Not Presented****: Models for Upper MG Loss Score, Lower MG Loss Score, MG Secretion Expressibility Score, and Upper Eyelid Meibum Quality were either not statistically significant (P>0.05) or could not be reliably estimated due to complete separation and are therefore not presented.
****⁽ᵐ⁾ Marginal Model Fit****: The overall model for CFS Score demonstrated a trend toward significance (P=0.051).
